# Tapping into neural resources of communication: formulaic language in aphasia therapy

**DOI:** 10.3389/fpsyg.2015.01526

**Published:** 2015-10-20

**Authors:** Benjamin Stahl, Diana Van Lancker Sidtis

**Affiliations:** ^1^Brain Language Laboratory, Department of Philosophy and Humanities, Freie Universität BerlinBerlin, Germany; ^2^Department of Communicative Sciences and Disorders, New York UniversityNew York, NY, USA

**Keywords:** formulaic language, left-hemisphere stroke, aphasia, apraxia of speech, Melodic Intonation Therapy, Intensive Language-Action Therapy, Constraint-Induced Aphasia Therapy, post-stroke depression and anxiety

Decades of research highlight the importance of formulaic expressions in everyday spoken language (Vihman, 1982; Wray, 2002; Kuiper, 2009). Along with idioms, expletives, and proverbs, this linguistic category includes conversational speech formulas, such as “You've got to be kidding,” “Excuse me?” or “Hang on a minute” (Fillmore, 1979; Pawley and Syder, 1983; Schegloff, 1988). In their modern conception, formulaic expressions differ from newly created, grammatical utterances in that they are fixed in form, often non-literal in meaning with attitudinal nuances, and closely related to communicative-pragmatic context (Van Lancker Sidtis and Rallon, 2004). Although the proportion of formulaic expressions to spoken language varies with type of measure and discourse, these utterances are widely regarded as crucial in determining the success of social interaction in many communicative aspects of daily life (Van Lancker Sidtis, 2010).

The unique role of formulaic expressions in spoken language is reflected at the level of their functional neuroanatomy. While left perisylvian areas of the brain support primarily propositional, grammatical utterances, the processing of conversational speech formulas was found to engage, in particular, right-hemisphere cortical areas and the bilateral basal ganglia (Hughlings-Jackson, 1878; Graves and Landis, 1985; Speedie et al., 1993; Van Lancker Sidtis and Postman, 2006; Sidtis et al., 2009; Van Lancker Sidtis et al., 2015). It is worth pointing out that parts of these neural networks are intact in left-hemisphere stroke patients, leading to the intriguing observation that individuals with classical speech and language disorders are often able to communicate comparably well based on a repertoire of formulaic expressions (McElduff and Drummond, 1991; Lum and Ellis, 1994; Stahl et al., 2011). An upper limit of such expressions has not yet been identified, with some estimates reaching into the hundreds of thousands (Jackendoff, 1995).

The above literature suggests that formulaic expressions may be viewed as a valuable resource in speech-language therapy. However, surprisingly little is known about their potential impact on the success of popular programs in clinical rehabilitation. The current opinion paper seeks to address this matter by outlining the contribution of formulaic expressions to seminal approaches in recovery from speech and language disorders after stroke.

## Utterance-oriented approaches: Music-based rehabilitation programs

According to analytical language philosophy and communicative-pragmatic theory, the meaning of an utterance emerges from its ordinary use by performing so-called “speech acts,” such as greeting a person (Wittgenstein, 1953; Austin, 1962; Searle, 1969; Horn and Ward, 2008). Adopting this idea for clinical rehabilitation, treatment programs in speech-language therapy should be grounded in behaviorally relevant situations that enable patients to benefit from a range of communicative features, including the turn-taking structure underlying everyday conversation (Pulvermüller, 1990). For example, the speech act of greeting offers the conversation partner a number of possibilities to respond—typically by using individual sets of formulaic expressions, such as “Good to see you,” “How's it going?” or “Long time no see.” One may claim that incorporating this turn-taking structure in speech-language therapy does not provide any added value for the outcome of the treatment. If this is true, the training of formulaic expressions in communicative-pragmatic context should be as successful as exercises that focus on articulatory quality of the same utterances, regardless of their social function. However, it remains questionable how effective such utterance-oriented approaches are in improving everyday language abilities over and above articulatory quality of trained expressions.

Prominent examples of utterance-oriented approaches in speech-language therapy are, in some respect, music-based rehabilitation programs, among them a treatment known as Melodic Intonation Therapy (Albert et al., 1973). The treatment protocol requires persons with non-fluent aphasia to produce sentences and phrases in different modalities, including singing and rhythmic sprechgesang (Helm-Estabrooks et al., 1989). While the higher difficulty levels of the protocol encourage the use of grammatical utterances, the lower levels involve formulaic expressions, such as “I am fine,” “How are you?” or “Thank you.” Although most of these expressions may occur naturally in a conversation, their repetitive training does not meet the criteria of communicative-pragmatic speech-language therapy. Among other caveats, Melodic Intonation Therapy does not benefit systematically from the turn-taking structure underlying everyday conversation in the training sessions. This may limit the transfer of trained sentences and phrases into real life, a goal of primary importance in clinical practice.

In line with this view, randomized controlled trials on Melodic Intonation Therapy should not consistently reveal generalized effects on standardized aphasia test batteries, even if the sample of trained sentences and phrases is relatively large (cf. van der Meulen et al., 2014; van der Meulen, 2015; for evidence on model-oriented approaches in speech-language therapy, see Brady et al., 2012). Nonetheless, music-based rehabilitation programs have been demonstrated to directly benefit the production of trained expressions in individuals with chronic non-fluent aphasia and apraxia of speech (Wilson et al., 2006; Stahl et al., 2013; Zumbansen et al., 2014). One may argue that the reported progress in the production of such expressions depends, at least in part, on increased activity in right-hemisphere neural networks engaged in the processing of formulaic language, especially when considering the repetitive character of the training (cf. Berthier et al., 2014). If this notion is correct, it would help to explain conflicting results from neuroimaging studies, indicating either left perilesional or right frontotemporal reorganization of language in patients treated with Melodic Intonation Therapy (Belin et al., 1996; Schlaug et al., 2008, 2009; Vines et al., 2011). Future trials will hopefully determine whether or not these discrepant findings arise from different degrees of formulaicity in the experimental tasks (cf. Stahl and Kotz, 2014).

## Communicative-pragmatic approaches: Therapeutic language games

Communicative-pragmatic rehabilitation programs for individuals with aphasia aim at training verbal expressions in behaviorally relevant settings, so-called “language games” (Davis and Wilcox, 1985; Pulvermüller and Roth, 1991; Bastiaanse and Prins, 1994). Based on a variety of utterances, patients are invited to communicate with others by performing different types of speech acts, such as requesting objects from a person. Importantly, the turn-taking structure of language games offers the conversation partner a number of possibilities to respond, including a series of formulaic expressions (“Here you are,” “You're welcome,” “I'm sorry,” “Too bad,” “Pardon me?”). In contrast to utterance-oriented approaches, language games focus less on articulatory quality of sentences and phrases rather than on their suitability in communicative-pragmatic context. One may therefore claim that such approaches should, in principle, be effective in improving everyday language abilities over and above articulatory quality of trained expressions.

Prominent examples of communicative-pragmatic approaches are clinical language games, including a treatment known as Intensive Language-Action Therapy (cf. Constraint-Induced Aphasia Therapy; Difrancesco et al., 2012). The treatment protocol requires up to three individuals with aphasia and a therapist to obtain picture cards from each other, such as by making verbal requests. Utterances are combined with manual actions by handing over the requested card to other players. Depending on the availability of picture cards, the players use adequate sets of formulaic expressions to indicate whether a request was accepted (“Here you are,” “Thank you,” “You're welcome”), rejected (“I'm sorry,” “No problem,” “Too bad”) or unclear (“Pardon me?”). That is, the repetitive interaction with formulaic expressions benefits from the rich turn-taking structure underlying everyday conversation, with possible implications on the success of the language game.

There is indeed ample evidence from randomized controlled trials suggesting that Intensive Language-Action Therapy induces generalized effects on standardized aphasia test batteries (Pulvermüller et al., 2001; Meinzer et al., 2005, 2007; Berthier et al., 2009). Although several elements included in the program are likely to contribute to this finding, the use of formulaic expressions may particularly account for the practicability of communicative-pragmatic approaches, allowing patients to tap into right-hemisphere language resources in the training sessions. Interestingly, neuroimaging studies have revealed either left perilesional or right frontotemporal functional reorganization in patients treated with Intensive Language-Action Therapy (Meinzer et al., 2004, 2008; Pulvermüller et al., 2005; Breier et al., 2006, 2009; MacGregor et al., 2014; Mohr et al., 2014; Barbancho et al., 2015). Future trials may help to clarify to what extent these results depend on increased activity in neural networks supporting formulaic language.

## Possible impact on motivation, well-being, and quality of life

Individuals with left-hemisphere brain damage often experience a sudden inability to engage in communication with others based on propositional, grammatical utterances. This loss of social interaction skills may be one reason for the high prevalence of severe psychopathological symptoms in the first year following acquired brain injury (cf. Lewinsohn, 1974). Notably, almost half of the patients suffer from post-stroke depression or anxiety during this period of time (Kauhanen et al., 1999; Fleminger et al., 2003; Schöttke and Giabbiconi, 2015). While antidepressant medication is an option for most patients with speech and language disorders, classical forms of psychotherapy remain challenging due to constrained verbal expression and comprehension.

A number of approaches in psychotherapy seek to identify and activate resources in order to overcome cognitive-affective distress (Priebe et al., 2014). Adopting this goal for clinical rehabilitation after stroke, formulaic expressions frequently remain one of the few resources available to communicate for patients with left-hemisphere brain damage. However, patients are commonly unaware of their ability to perform sets of formulaic expressions correctly. Using these utterances in therapy may therefore play a key role in compensating for loss of social interaction, with a possible beneficial influence on motivation, subjective well-being, and quality of life (Doering et al., 2011; Hilari et al., 2012; Kuenemund et al., 2013). Although anecdotal evidence confirms the positive non-linguistic effects of formulaic language in stroke patients, this hypothesis has not been studied experimentally.

We wish to emphasize that current programs in speech-language therapy differ considerably in how they take advantage of formulaic expressions, drawing on neural resources of communication, to support social interaction. As discussed previously, utterance-oriented approaches focus mainly on articulatory quality in the training sessions. In contrast, communicative-pragmatic approaches benefit from the rich turn-taking structure underlying everyday conversation, thus encouraging the use of formulaic expressions in natural settings. We believe that methods relying on preserved language abilities in contexts of social interaction may have a substantial impact on recovery from cognitive-affective distress, especially in persons with concomitant post-stroke depression and anxiety—a claim yet to be confirmed empirically.

## Open questions for future research

A growing body of research provides compelling evidence for the contribution of right-hemisphere cortical and bilateral subcortical neural systems to the production and comprehension of formulaic language. These data are consistent with the notion that the efficacy of prominent approaches in speech-language therapy is, to some degree, dependent on the intensive use of formulaic expressions. However, it is still poorly understood how exactly the language system of the damaged brain benefits from neural resources associated with formulaic expressions. There are, in fact, a range of neurophysiological scenarios that may account for descriptions of preserved language skills in clinical rehabilitation.

According to Hebbian learning, the synchronous firing of cell assemblies is likely to strengthen the neural connectivity between them, even if they are located in distributed areas of the brain; in other words, “cells that fire together, wire together” (Hebb, 1949). This neurobiological model may be appropriate in addressing three fundamental questions in future research: (i) Does intensive training of formulaic expressions stimulate neural activity in right-hemisphere cortical and bilateral subcortical language circuits? (ii) Does the combined training of grammatical utterances and formulaic expressions lead to functional reorganization in the interplay of left perilesional and intact right-hemisphere language networks? (iii) Does this bilateral neural interplay affect treatment-induced generalized effects observed on standardized aphasia test batteries?

With this article, we wish to increase the awareness for neural resources of communication in the treatment of left-hemisphere stroke patients. We readily acknowledge that the examples included may only be the “tip of the iceberg.” Based on our experience, the ability to use formulaic expressions is often well documented in clinical practice, commonly under a variety of different terms. However, the possible influence of such expressions on the outcome of speech-language therapy frequently remains unnoticed. Uncovering the behavioral and neural dynamics of formulaic expressions may therefore be crucial in identifying and activating resources of communication more systematically. This may help to improve the success of current attempts to promote recovery from speech and language disorders and cognitive-affective distress after stroke.

### Conflict of interest statement

The authors declare that the research was conducted in the absence of any commercial or financial relationships that could be construed as a potential conflict of interest.
